# Synthesis and Acoustic Study of a New Tung Oil-Based Polyurethane Composite Foam with the Addition of Miscanthus Lutarioriparius

**DOI:** 10.3390/polym11071144

**Published:** 2019-07-04

**Authors:** Yangjie Ji, Shuming Chen, Yabing Cheng

**Affiliations:** 1 State Key Laboratory of Automotive Simulation and Control, Jilin University, Changchun 130022, China; 2 School of Mechanical and Aerospace Engineering, Jilin University, Changchun 130022, China

**Keywords:** bio-based polyurethane foam, Filler, miscanthus lutarioriparius, acoustic performance, mechanical properties

## Abstract

Polyurethane foam is commonly used in the automobile industry due to its favorable acoustic performances. In this study, a new tung oil-based polyurethane composite foam (TOPUF) was prepared by a one-step method. Different forms and contents of miscanthus lutarioriparius (ML) were used in TOPUF for improving acoustic performance. Polyurethane foams were characterized by means of Fourier transform infrared and SEM. The acoustic properties and mechanical properties of TOPUF, obtained with ML, were determined and compared with pure petroleum-based polyurethane foam. The results illustrate that the modification of TOPUF with the ML has a positive effect on the acoustic and mechanical properties in comparison to the unmodified foam. TOPUF obtained with ML powders has better acoustic performance than that obtained with ML strips. The optimum acoustic performance is achieved at the filler content of 0.3 wt%. The average sound absorption coefficient and transmission loss can reach 0.518, and 19.05 dB, respectively.

## 1. Introduction

Fossil resources are used for various applications, including energy generation, running transportations, polymer, and plastic synthesis [1]. Fossil resources are non-renewable, and they also produce numerous harmful substances, such as benzene and hydrogen sulfide. With the advancement of society and the increasing awareness of environmental protection, there is considerable focus on the use of alternative renewable energy sources. Polyurethane foam (PUF) is widely used as an acoustic material, due to its superior sound-absorbing properties, vibration damping, and robustness in automobile industry [2,3]. The pure petroleum-based polyurethane foam (PPUF) consumes numerous fossil resources. In addition, it causes great damage to the environment.

Raw materials for synthetic vegetable oils, include common soybean oil, castor oil, palm oil, etc. Sonnenschein et al. [4] prepared a flexible PUF by means of soybean oil-based polyols and toluene diisocyanates. Bonnaillie et al. [5] used Epoxidized soybean oil to produce a thermosetting elastic foam of high mechanical properties through a carbon dioxide foaming process. Spontón et al. [6] found that biodegradability of flexible PUF is greatly improved after being modified by castor oil. The effect of hydroxyl in the polyols on mechanical properties was studied when flexible PUF was prepared with Rapeseed oil [7,8,9]. Marcovich et al. [10] and Prociak et al. [11] mixed palm oil polyols and polyether polyols to prepare polyurethane foams. Zhang et al. [12] investigated the mixed situation of soy-castor oil-based polyols and petroleum-based polyols to evaluate the miscibility. 

Tung oil is a suitable raw material to produce high hydroxyl content polyols in the synthesis of PUF. Tung oil is a renewable resource. It can be easily extracted because tung tree is widely distributed in China. In addition, tung oil offer a priori of possibilities for biodegradation [13]. Among various available oil, tung oil is a conjugated drying oil. Tung oil has a high iodine value and contains a conjugated triene on the triglyceride molecule [14]. Furthermore, tung oil has the advantages of faster drying time, high corrosion resistance, and high hardness as a result of high levels of unsaturation. Therefore, tung oil is an important industrial oil with a wide range of applications in paints, ships, and materials [15]. To the best of our knowledge, very few research studies have been done on tung oil-based flexible polyurethanes foam (TOPUF), especially their acoustic properties.

There are numerous modification methods to improve the performance of PUF, including physical modification methods (alloying, filling, etc.) and chemical modification methods (interpenetrating polymer network, copolymerization grafting crosslinking, etc.) [16,17,18,19,20]. Adding filler is a common modification method because it is simple and relatively inexpensive. From the perspective of properties, fillers are divided into organic and inorganic fillers. They contain granules, flakes, and fibrous fillers in terms of morphology. Sung et al. [21] investigated the morphological and physical properties of PUF with addition of inorganic fillers including Talc, Zinc Borate, and Aluminum Hydroxide. Chen et al. [22] added bamboo leaf particles to the PUF to ameliorate the acoustic properties. Santos et al. [23] described the influence of the concentration of lignin as a filler of PUF for crude oil sorption. Zhang et al. [24] prepared castor oil-based PUF by self-rising method with soy protein isolate as reactive reinforcing filler. Merlini et al. [25] used graphene nanoplatelets, expanded graphite, multiwall carbon nanotubes, and carbon black filler in fabricating thermosetting PUF for improving mechanical property.

The use of organic materials to modify PUF takes full advantage of natural resources. Miscanthus lutarioriparius (ML) is widely distributed in the middle and lower reaches of the Yangtze River in China. It is considered to be one of the most promising second-generation biomass resources [26]. Miscanthus lutarioriparius contains high lignocelluloses and carbohydrate with 50% content of fiber. It can be cultivated under different soil and climatic conditions [27]. Nowadays, it is often used in paper and fiberboard because of its excellent fiber quality. ML is also suitable as an organic filler to be added to polymers due to its stable structure and high cellulose content. So far, as far as we know, ML has not been used as a kind of filler for PUF in current researches. In addition, there are few research results on the effects of fillers on the acoustic properties of PUF.

In this study, we reported the effect of ML filler on acoustic and mechanical properties of TOPUF. Firstly, tung oleic acid-based polyol (TOAP) was used to replace a part of petroleum materials to prepare TOPUF. An amount of 0.3–1.5 wt% of ML was loaded into TOPUF to improve the acoustic properties, especially in low frequency rang. Miscanthus lutarioriparius in strip and powder form were used to prepare TOPUF with addition of ML (TOPUFL). Then, PUF were characterized by means of Fourier transform infrared (FTIR) and SEM. Then, we determined the cell morphology, acoustic parameters, and mechanical properties of TOPUFL. Finally, the TOPUFL was compared with TOPUF and PPUF.

## 2. Materials and Methods

### 2.1. Materials

Polyurethane foam is synthesized with polyols and Methylenediphenyl diisocyanate (MDI). The polyols include polyether polyols 3630 (OH-value: 21–27 mg KOH/g), 330N (OH-value: 36 mg KOH/g), TOAP (OH-value: 430–470 mg KOH/g). A33 selected as the amine catalysts is composed of 33% triethylene diamine solution. The tyriethanolamine (TEA) plays a dominant role in controlling the mechanical properties of the foam. Silicone was selected as the foam stabilizer and surfactant. Deionized water (DIW) was used for blowing agent. Miscanthus lutarioriparius were added as a filler to the PUF. While, 5 wt% NaOH solution is as a treatment solution for filler. The names, roles and supplier of the materials used are listed in Table 1. 

### 2.2. Experiment Design

**Figure 1:**
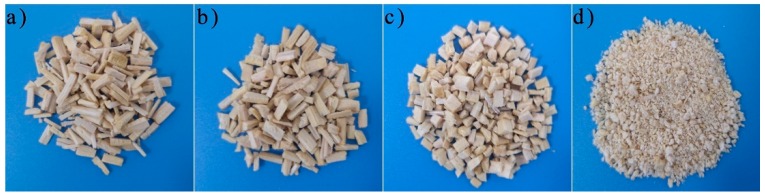
The shape of 10 mm (**a**), 7 mm (**b**), 4 mm (**c**) miscanthus lutarioriparius (ML) strips and ML powders (**d**).

Miscanthus lutarioriparius was cut into strips of 10 mm, 7 mm, and 4 mm. In addition, part of ML was stirred into powders in a shredder at a rate of 22,000 rpm for 30 s. The size of the ML powders are between 0.05 mm and 0.1 mm. The shape of the filler is shown in Figure 1. Then, ML was immersed in the 5 wt% NaOH solution for 20 min. After that, ML was taken out and washed with DIW until the pH of ML surface is 7. At last, the ML became fillers after being dried at 80 °C for 24 h in a dry box. 

**Figure 2:**
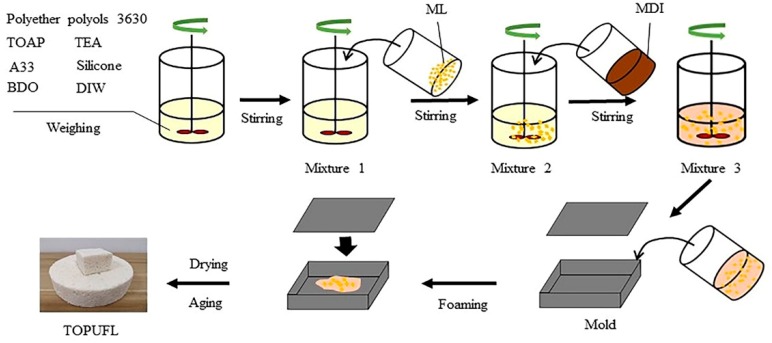
The synthesis process of TOPUFL.

The one-step polymerization process was selected to prepare the TOPUF. Tung oleic acid-based polyol can be obtained by epoxidation and hydrogenation of tung oil. Then, TOAP and polyether polyols 3630 are fully mixed. The mixture can react further with MDI to obtain TOPUF. The TOPUF is a network polymer. The raw materials and synthesis process of TOPUFL are shown in Table 2 and Figure 2. Firstly, each component was weighed on an electronic balance. Secondly, materials except for MDI, were mixed at 1600 rpm for 20 s and then at 1000 rpm for 20 s. Miscanthus lutarioriparius was added to Mixture 1. Finally, MDI was added to this Mixture 2, and the whole Mixture 3 was stirred at 1300 rpm for 8 to 10 s. After the Mixture 3 was poured into the mold rapidly, the foam was hold at 50 °C for 60 min in a drying oven. 

**Figure 3:**
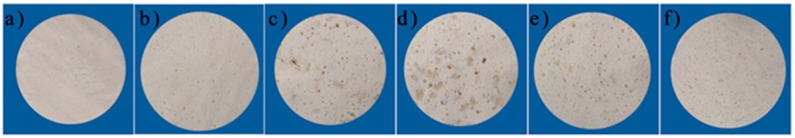
The first set of test samples. Pure petroleum-based polyurethane foam (PPUF) (**a**); TOPUF (**b**); TOPUF adding 10 mm (**c**), 7 mm (**d**), 4 mm (**e**) ML strips and ML powders (**f**).

**Figure 4:**
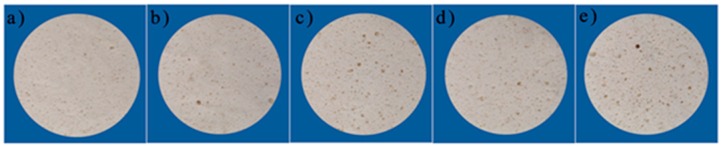
The second set of test samples. TOPUF adding 0.3 wt% (**a**), 0.6 wt% (**b**), 0.9 wt% (**c**), 1.2 wt% (**d**) and 1.5 wt% (**e**) ML powders.

The experiment is divided into two stages in order to study the effect of fillers of different shapes. Miscanthus lutarioriparius strips of 10 mm, 7 mm, 4 mm and powders are added to the TOPUF. Their qualities are all controlled at 0.9 g. Through acoustic testing, we found that the acoustic performance of TOPUF with the addition of ML powders is the best. Therefore, in the second stage, we compared the performance of TOPUFL when the filler was 0.3–1.5 wt%. Figure 3 and Figure 4 show the sample foams used for testing.

### 2.3. Measurement Method

The FTIR spectra of PUF were recorded over the range of 4000–400 cm^−1^ by a Nexus670 FTIR spectrometer (Thermo Electron Corporation, Madison, WI, USA). The morphology of the samples was characterized by field emission scanning electron microscopy (FESEM; ZEISS EVO18). The sound absorption coefficient and transmission loss test were performed using the SCS90AT two-microphone impedance tube device (SCS, Padova, Italy). The test was performed at room temperature (20 °C) under a relative humidity of 65%. Sound absorption properties of porous materials are mainly measured by the sound absorption coefficient [28]. The average sound absorption coefficient ($\bar{\alpha }$) could be calculated by

(1) $$\bar{\alpha }=\frac{\alpha_{125}+\alpha_{250}+\alpha_{500}+\alpha_{1000}+\alpha_{2000}+\alpha_{4000}}{6}$$

where $\alpha_{125}\sim \alpha_{4000}$ represent the sound absorption coefficients at 125 Hz, 250 Hz, 500 Hz, 1000 Hz, 2000 Hz, and 4000 Hz, respectively. Each material was measured six times. The transmission loss representing the attenuation of the sound energy can evaluate the sound attenuation effect of the material. The Loss of sound power transmission (TL) was calculated by:

(2) $$TL=10\mathrm{lg}(P_{1}^{2}/P_{2}^{2})$$

where $P_{1}$ and $P_{2}$ represent the sound pressure at the entrance and exit of the sample, respectively. Each material was measured six times. The average transmission loss ($\bar{TL}$) is the average value of the transmission loss at 1/3 octave band of 100–4000 Hz. It could be calculated by:

(3) $$\bar{TL}=\frac{TL_{100}+TL_{125}+\cdots +TL_{3125}+TL_{4000}}{17}$$

where $TL_{100}$~$TL_{4000}$ represent the transmission loss at frequencies of 100 Hz, 125 Hz, …, 4000 Hz, respectively (1/3 octave band of 100–4000 Hz). The flow resistance apparatus was devised according to the requirements of the standard ASTM C522-03 (2009). An air pressure difference (Δp) created by a steady airflow rate (Q) crossing the sample, with cross sectional area (S) and specific thickness (d) could be acquired by the apparatus. The airflow resistivity (σ) could be calculated by:

(4) σ=Δp×S/(Q×d)

Porosity “P” is the percentage of pore volume in bulk materials to the total volume of materials under natural conditions. The porosity (P) could be calculated by:

(5) $$P=\frac{V_{0}-V}{V_{0}}\times 100\%$$

where $V_{0}$ represents apparent volume. V represents absolutely compact volume. The porosity apparatus was devised according to the measurement method of pressure/mass [29]. The sample is a disc with a diameter of 10 cm. Mechanical properties of PUF were performed using electronic universal testing machine (TM 2101) (Lixian Instrument Co., Ltd., Dongguan, China). The sample, that was subjected to the compression test, was cut into a rectangular parallelepiped. The length and width of the rectangular parallelepiped are 30 mm, and the height is 20 mm. Tensile properties of PUF was measured according the ASTM D638. This sample is dumbbell shaped of 35 mm thickness.

## 3. Results and Discussion

### 3.1. FTIR Analysis

**Figure 5:**
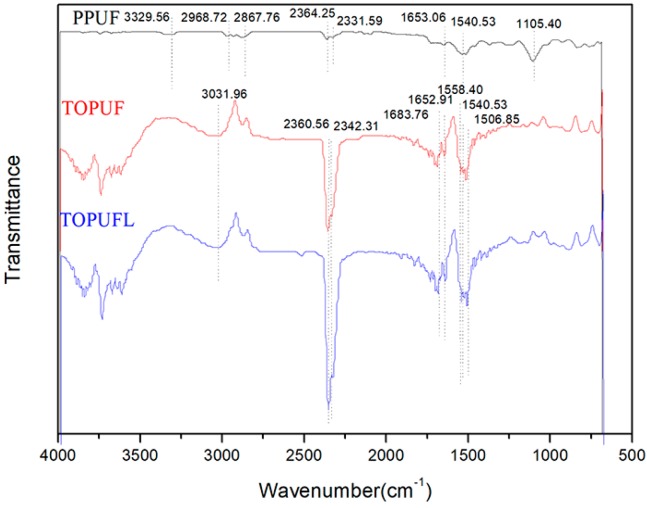
FTIR spectrums of different polyurethane foams.

The FTIR spectrums of the TOPUF and TOPUFL are shown in Figure 5. The PPUF is selected as a comparison. As for PPUF, Polyurethane is formed, on account of the peak 1504.53 cm^−1^ (N–H bending). The appearance of peak 1105.40 cm^−1^ (C–O–C stretching) indicates the generation of an ether bond. PPUF is a polyether polyol type polyurethane. As for TOPUF and TOPUFL, the feature absorptions are at 3031.96 cm^−1^ (asymmetrical C–H stretching), 2360.56–2342.31 cm^−1^ (CO_2_ in the air), 1683.76–1652.91 cm^−1^ (C=O stretching), 1558.40 cm^−1^ (C–N Stretching vibrations), 1540.53 cm^−1^ (N–H bending), 1506.85 cm^−1^ (Benzene ring). The characteristic peaks of TOPUF and TOPUFL are the same. It indicates that the added filler did not react with the starting material to form a new functional group. Compared with the characteristic peak of PPUF, the TOPUFL has something new. Massive carbamate (–NHCOO–) groups are generated by the appearance of C–N Stretching vibrations. More benzene rings are formed according to the prominent peak of 1506.85 cm^−1^. Tung oleic acid-based polyol reacts with isocyanate to obtain a new product according to FTIR spectrum of the TOPUF. The characteristic absorption peaks do not appear at 2260–2280 cm^−1^. This indicates that there are no free isocyanate (–NCO) groups left, and the isocyanate reacts completely.

### 3.2. Morphological Characterization

**Figure 6:**
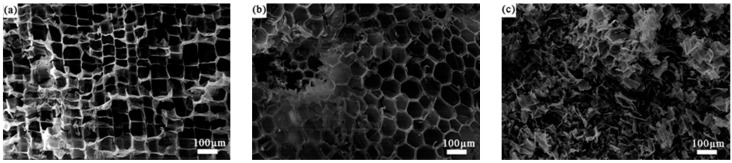
SEM images of radial section (**a**), tangential section (**b**), and powder of ML (**c**).

Figure 6 shows the morphological characterization of ML. The basic unit of ML is the cell. This cell is box-shaped on the radial section. Each box is 80 to 100 μm in width. The cells present an approximate hexagon on the tangential section. It can be seen from (a) and (b) in Figure 6 that the cells of ML are closed hexagonal prisms. Due to these closed hexagonal prisms, the ML contains many independent small spaces and is an excellent energy absorbing material. As shown in Figure 6c, numerous wrinkles are formed after the ML is broken by a shredder. Each pillar of the regular hexagonal unit is connected to the wall of three cells.

**Figure 7:**
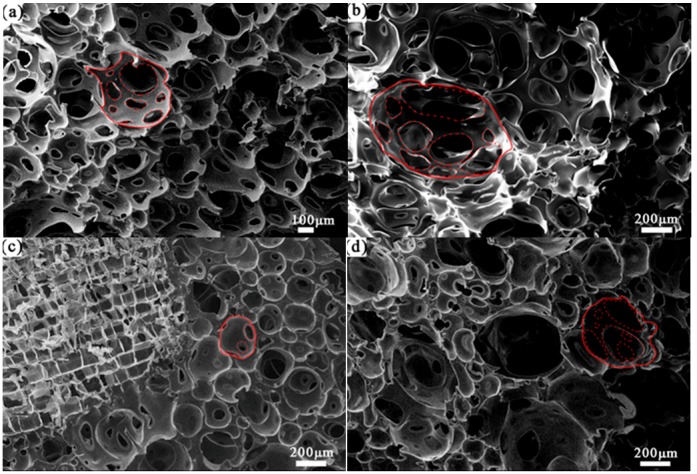
SEM images of PPUF (**a**), TOPUF (**b**), TOPUF with ML strips (**c**) and powders (**d**).

As is shown in Figure 7a, the interior of PPUF is a generally uniform open cell foam. Figure 7b shows that the cavity sizes of cells are inconsistent due to the addition of vegetable oil. As can be seen from Figure 7c, the combination of the ML strips and TOPUF is close because no large holes are created on the bonding surface. Figure 7d, shows that ML powders can be well integrated into TOPUF. In addition to the cavities of varying sizes, this material creates large voids. A large number of small cavities are gathered together with the filler. There is a large tension between the small cavities to form a thick inner wall. The inner wall of the areas without plenty of small cavities thin or even break up to form large holes. The pore and cavity sizes of different types of PUF are summarized in Table 3. Pore sizes and cavity sizes of TOPUF are both larger than PPUF. The pore and cavity sizes of the PUF have changed after adding ML strips filler. The cavity and cell sizes are reduced for TOPUF with ML strips. Miscanthus lutarioriparius strips have a certain compressive force on the surrounding polyurethane. Therefore, the polyurethane cavity becomes small. Miscanthus lutarioriparius powders have not the same compression force as ML strip because the volume of ML powders is relatively small.

**Figure 8:**
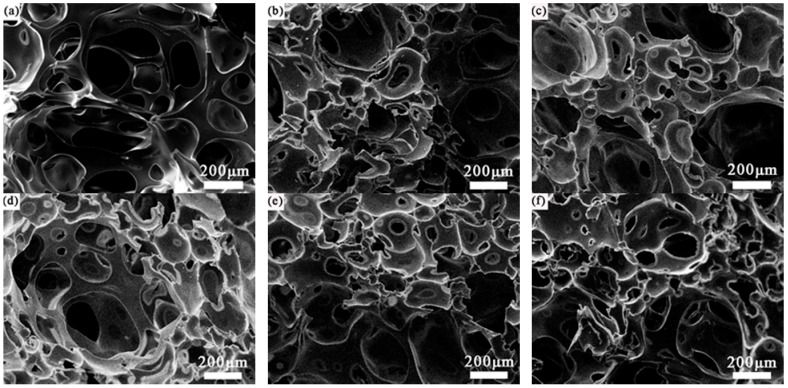
SEM images of TOPUF (**a**), TOPUF with 0.3 wt % (**b**), 0.6 wt % (**c**), 0.9 wt % (**d**), 1.2 wt % (**e**), and 1.5 wt % (**f**) ML powders.

Effects of different amounts of ML powders are studied on the morphology of TOPUF. As is shown in Figure 8, 0–1.5 wt% powders are added to the TOPUF. The pore and cavity sizes of the TOPUFs with the addition of different ML powders are counted in Table 4. As the content of ML powder increases, the size of the cavity size will decrease. Additionally, the number of small cavity sizes will increase. In general, the morphology of TOPUFs with the inclusion of ML powders have common features. They are all composed of cavities with large differences in size. The small cavities exhibit the characteristics of partial area aggregation. Miscanthus lutarioriparius powders have agglomeration effect on TOPUF matrix. The viscosity of the TOPUF is extremely high. 

**Figure 9:**
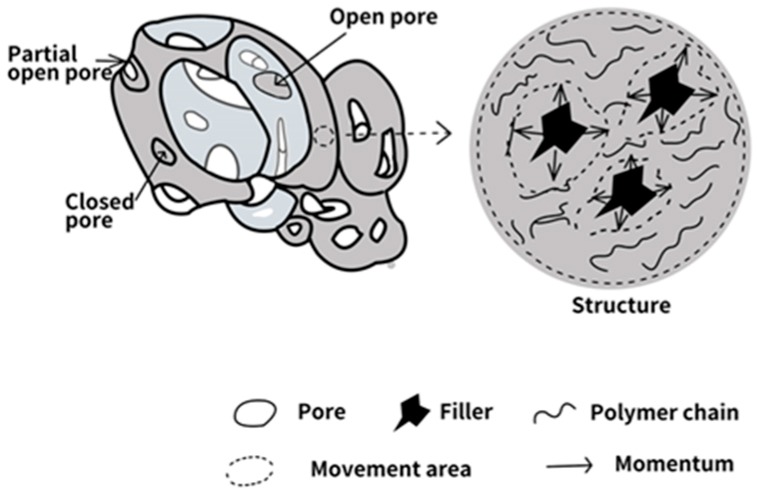
Schematic illustration of mechanical damping effects of TOPUF adding ML powders under sound waves.

According to the SEM images of TOPUF with ML powders, Figure 9 shows the damping effect of adding ML powders to TOPUF. There are three types of holes on the outside cavity: Open pore, partial pore, and closed pore. Sound waves can pass through open pores and partial open pores, while acoustic waves can also be scattered and reflected at the closed pore and cavity walls. The energy of the sound is dissipated by scattering and reflecting. It can further improve the sound absorption efficiency. The transmission path of sound waves in the foam is prolonged through the scattering and reflection of sound waves. The more scattering and reflection the sound waves have, the greater the energy loss will be. The ML powders also perform a damping motion in the movement area of the cavity enhancing the dissipation of the sound. Due to the three-piece structure of ML powder, it can have a good dissipative effect on sound waves in various directions.

### 3.3. Acoustic Properties Analysis

Acoustic performance includes, sound absorption performance and sound insulation performance. Sound absorption performance mainly comes from two sound absorption mechanisms: Friction between air molecules and acoustic energy and the collision of sound waves with polyurethane matrix [30]. The sound absorption properties of materials are mainly affected by porosity, structural factor, flow resistance, bulk density, and thickness [31]. Sound insulation performance mainly comes from the blocking effect of PU matrix on sound waves. The higher the unit density of the material, the better the corresponding sound insulation effect [32].

#### 3.3.1. Acoustic Properties Analysis of TOPUF Adding Different Forms of ML

**Figure 10:**
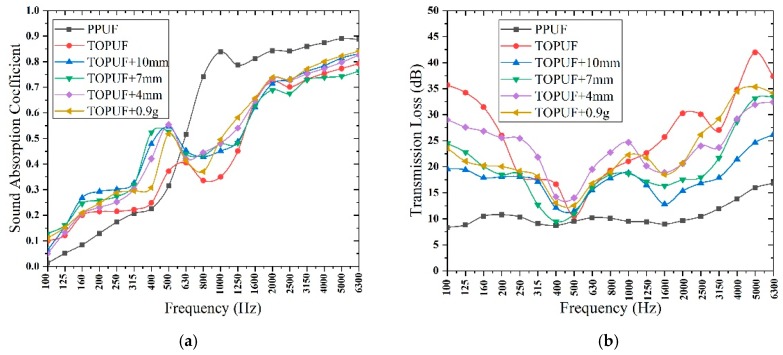
Sound absorption coefficient (**a**) and transmission loss (**b**) of TOPUF adding different forms of ML.

The sound absorption coefficient and transmission loss of TOPUF, including different forms of ML, are shown in Figure 10. Table 5 shows the average sound absorption and transmission loss of TOPUF, including different forms of ML. The quality of ML strips and powders is controlled at 0.9 g. The length of ML strips is 10 mm, 7 mm, and 4 mm, respectively. It can be found that PPUF has favorable sound absorption performance at medium frequency (500–2000 Hz) and high frequency (above 2000 Hz). However, PPUF shows unsatisfactory sound absorption behavior at low frequency (100–500 Hz) and sound insulation performance over the entire frequency range (100–6300 Hz). Different from PPUF, TOPUF has excellent sound absorption performance in the low frequency range and sound insulation performance in the whole frequency range. The sound absorption coefficients of low and intermediate frequency performance of TOPUF improved, due to the addition of ML strips and powders. The value of average sound absorption coefficient of TOPUFL is in the range of 0.478 to 0.492. For TOPUF with different lengths of ML strips, the sound absorption coefficient does not appear large variation when the amount of addition is constant. The average transmission loss of TOPUF is 24.546 dB. Transmission loss of the TOPUF is reduced after adding ML. As shown in Figure 8, the cavities and pores of the TOPUF are larger and more uneven than that of PPUF. Excessive pore size will make it easy for sound waves to pass through which result in less sound absorption. After the filler is added to TOPUF, the number of cavities increases, the size of the pores decreases, and the areal density reduces. The uniformity of the combination of ML powders and TOPUF is better than that of ML strips. A material can be considered as a good sound-absorbing material if its average sound-absorbing coefficient exceeds 0.5. As can be seen from Table 5, the acoustic performance of TOPUF adding ML powder is close to 0.5. At the same time, the average transmission loss of the TOPUF adding ML powder is 20.947 dB. In conclusion, TOPUF obtained with ML powders has more excellent acoustic performance than TOPUF obtained with ML strips.

#### 3.3.2. Acoustic Properties Analysis of TOPUF Adding Different Mass of ML Powders

**Figure 11:**
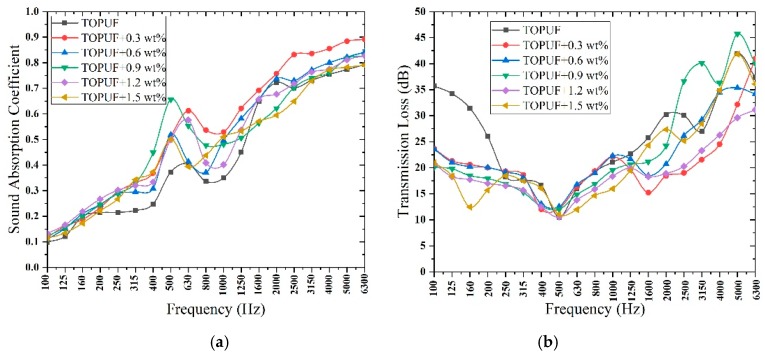
Sound absorption (**a**) and transmission loss (**b**) of TOPUF adding different mass of ML powders.

**Figure 12:**
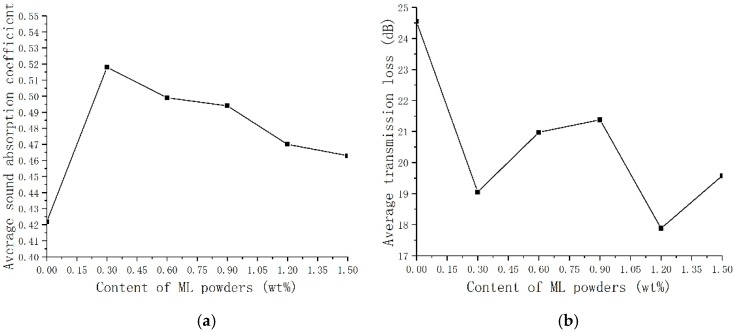
Average Sound absorption coefficient (**a**) and transmission loss (**b**) of TOPUF adding different mass of ML powders.

Sound absorption coefficient and transmission loss of TOPUF adding different masses of ML powders are shown in Figure 11. It can be found that the trend of sound absorption coefficient and transmission loss curves of these materials are consistent. The value of high frequency sound absorption coefficient is mostly concentrated around 0.7 to 0.8, and the highest can reach close to 0.9. Figure 12 shows the average sound absorption and transmission loss of TOPUF adding different masses of ML powders. The average sound absorption coefficient of the TOPUF adding the ML powders is concentrated between 0.463 and 0.518, which is much higher than TOPUF. The average transmission loss of TOPUF adding the ML powders is concentrated between 17.881 and 20.379 dB. Although it is lower than TOPUF, it is still a huge breakthrough compared with PPUF. As shown in Figure 8, small cavities formed, the size of pore decreased with the increasing of ML powders. 

**Figure 13:**
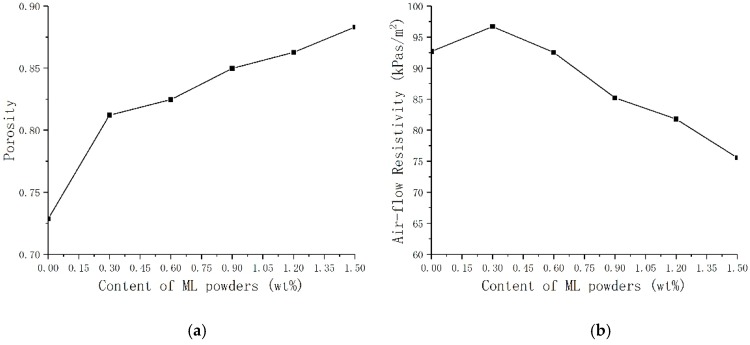
Porosity (**a**) and air-flow resistivity (**b**) of TOPUF adding different mass of ML powders.

The porosity and air-flow resistivity are used to assess the acoustic performances [33]. Sound waves can penetrate into the interior of the material through the voids inside the porous material. The greater the porosity, the further the sound waves can pass through. Low air-flow resistivity indicates that the material has less resistance to airflow, while too high air-flow resistivity indicates that the airflow is blocked [34]. If the flow resistance is small, the sound absorption capacity will become worse because of the decrease of the friction and viscous forces. The higher the flow resistance resistivity is, the better the low frequency sound absorption effect of the material can be obtained [35]. When the flow resistance resistivity is too high, the sound absorption capability will reduce due to the ability of air penetration decreases. Figure 13 shows the porosity and the air-flow resistance rate of TOPUF adding different masses of ML powders. The porosity of TOPUF increases, and the air-flow resistance rate decreases gradually as ML powders increase. At the beginning, the porosity of TOPUFL increases intensely. However, the exaltation of porosity is not obvious with the further increase of ML powders. The flow resistance rate decreased from 96.68 kPa/m^2^ to 75.55 kPa/m^2^. The 96.68 kPa/m^2^ does not exceed the optimum flow resistance of this material. The decrease in flow resistance rate has a strengthening effect on the sound absorption performance of the material than the increase in porosity. Combined with Table 5, we can find that the TOPUF has the highest sound absorption coefficient at the content of 0.3 wt%. 

**Figure 14:**
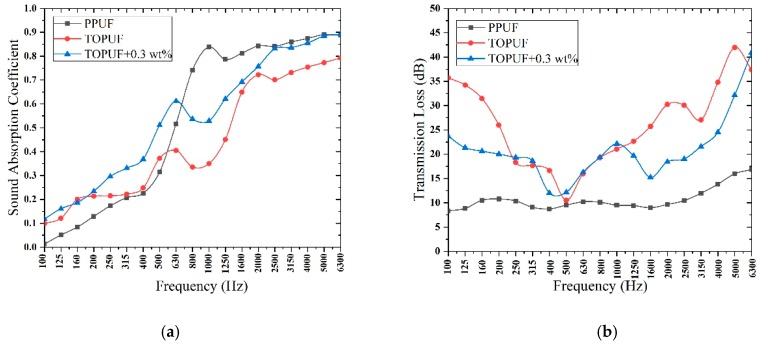
Comparison of sound absorption coefficient (**a**) and transmission loss (**b**) between different PUF.

As mentioned above, we can find that the sound absorption performance of TOPUF with 0.3 wt% ML powders is the best, and the performance of sound insulation performance under these circumstances is also favorable. Figure 14 shows the comparison of sound absorption coefficient and transmission loss between different PUFs. Compared with TOPUF, the sound absorption coefficient of TOPUF, including 0.3 wt% ML powders is higher completely. Although it is lower than PPUF at intermediate frequency, it is higher than PPUF at low frequencies and close to PPUF at high frequencies. The transmission loss of TOPUF adding 0.3 wt% ML powders is a little smaller than TOPUF. However, it is much higher than PPUF. The average sound absorption coefficient and transmission loss can reach 0.518 and 19.05 dB.

### 3.4. Mechanical Properties Analysis

**Figure 15:**
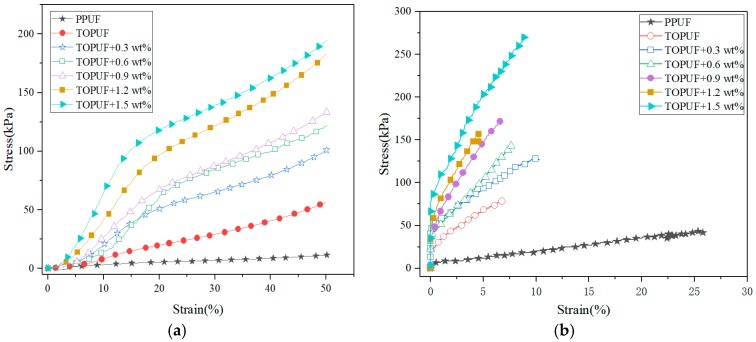
Stress-strain curves for compression (**a**) and tensile (**b**) of TOPUF adding different mass of ML powders.

The physical properties of the PUF applied for sound absorption materials should be considered to ensure long-term use. In general, the mechanical properties of PUF can be improved by adding fillers to PUF [21]. Compression strength can be considered as the direct response of the resisting force against various stresses. Tensile strength can be a measure of product durability under permanent load conditions. As it can be seen in Figure 15, the compression and tensile properties of TOPUF can withstand a much higher strength than PPUF. Additionally, it can be seen from Figure 15a, that the pressure that PPUF can bear is relatively small, while TOPUF adding ML powders is much stronger. The TOPUF adding ML powders is linearly elastic at a strain of about 15%. When the strain is more than 15%, the stress-strain curves change slowly. When it is around 35%, the stress increase will become severe, indicating that the cells begin to collapse. As is shown in Figure 15b, several sets of TOPUF materials can withstand much greater stress than PPUF. However, their elongation is relatively poor at break. 

Table 6 shows the comparison of mechanical properties of several PUF. Compressive strength is from what the PUF is being compressed to fifty percent of the statistics. We can see that the compressive strength can be increased from 56.67 to 194.56 kPa by adding ML powders. It exceeds the traditional PPUF greatly. Its breaking strength also increases from 78.33 to 273.33 kPa. The plasticity of TOPUF materials is not satisfactory because its elongation at break indeed has reduced to 9.51%. The reason for this phenomenon is that the addition of the filler increases the viscosity of the TOPUF. With the addition of ML powders, the material becomes harder, and its brittleness is also excellent, except the awful plasticity. The mechanical properties of the foams are primarily influenced by the size of the cells [36]. Plenty of small cavities are formed, due to the increase in the amount of ML powders. Small cavities formed with the ML powders (see Figure 8) can have more supporting walls and struts, and thus it resulted into the high compression strength. In addition, the ML powders act as reinforcement particles for the TOPUF to generate localized stresses [37]. The strength of the filler is higher than that of the surrounding PU matrix. Energy dissipation occurs for a growing fracture meets a filler particle in a reinforced polymer [38]. The number of ′barriers′ preventing fracture growth also increases as filler concentration increases. Therefore, the strength of the material will increase after the addition of the ML powders. The elongation was generally inversely proportional to the modulus. As the composite hardens, it became brittle and was prone to breaking without elongating much [39]. Considering the polyurethane as an acoustic packaging is mainly subjected to pressure, the TOPUF was modified with the ML powders has a great practicality in terms of mechanical properties.

## 4. Conclusions

A novel polyurethane acoustic material modified with ML of different forms (stripe and powder) and content (0.3–1.5 wt%) were prepared to improve acoustic performance in this paper. A noticeable improvement of sound absorption properties can be achieved by adding ML as fillers into PU foams. The results illustrate that the addition of ML has an influence on the size of the cavity, surface density, pores, as well as the wall thickness. The acoustic performance of TOPUF with ML powders is superior to ML strips on account of the uniformity of the combination of ML powder filler and TOPUF. It has been found that small cavities form, and the size of pores decreases with the increasing of ML powders. These structural changes affect the absorption and reflection of sound waves in the cavity. The results showed that TOPUF with 0.3 wt% of ML possess an optimal acoustic performance, whose average sound absorption coefficient and transmission loss can reach 0.518, and 19.05 dB, respectively. In addition, the porosity is 0.81, and the flow resistance rate is 96.68 kPa/m^2^. Moreover, the pressure resistance of ML will increase with the addition of ML powders. Its compression strength can be increased by three times compared with TOPUF. In contrast with PPUF, the TOPUFL has excellent low-frequency sound absorption performance, sound insulation performance, and mechanical properties. TOPUF, containing large numbers of biological components, are more environmentally friendly, and have a more comfortable odor. At the same time, TOPUF with the addition of ML, can make the full use of ML and reduces the waste of nature resources. To sum up, TOPUF adding ML can be a good acoustic packaging material with infinite application prospects. The results and method can be used as guidance for future design of acoustic materials. 

## Figures and Tables

**Table 1:** Major information of the material.

Name.	Role	Suppllier
3630	Polyether polyol	Jining Huakai Resin Company, Jining, China.
TOAP	Polyether polyol	Jining Huakai Resin Company, Jining, China.
330N	Polyether polyol	Jining Huakai Resin Company, Jining, China.
MDI	Isocyanate	Jining Huakai Resin Company, Jining, China.
A33	Catalyst	Guangzhou Yiju Chemical Company, Guangzhou, China.
TEA	Chain extender	Guangzhou Yiju Chemical Company, Guangzhou, China.
Silicone	Stabilizer and Surfactant	Guangzhou Yiju Chemical Company, Guangzhou, China.
DIW	Blowing agent	Laboratory extraction.
ML	filler	Songzhitao Department Store, Yueyang, China.
NaOH	treatment solution	Laboratory extraction.

**Table 2:** Foam formulation and design.

Component	Content of PPUF (g)	Content of TOPUF (g)	Content of TOPUFL (g)
TOAP	0	40.00	40.00
3630	40.00	60.00	60.00
330N	60.00	0.05	0.05
MDI	30.00	40.00	40.00
A33	1.00	2.00	2.00
BDO	0	3.00	3.00
TEA	3.00	0	0
Silicone	1.80	2.00	2.00
DIW	3.00	2.00	2.00
ML	0	0	0.45–2.25 (0–1.5 wt%)

**Table 3:** Sizes of pores and cavities in different polyurethane foam (PUF).

Name	Pore Size (μm)	Cavity Size (μm)
PPUF	159 ± 88	427 ± 192
TOPUF	318 ± 238	538 ± 358
TOPUF with ML strips	90 ± 62	180 ± 117
TOPUF with ML powders	250 ± 197	390 ± 260

**Table 4:** Sizes of pores and cavities in different TOPUFs.

Name	Pore Size (μm)	Cavity Size (μm)
TOPUF	318 ± 238	538 ± 358
TOPUF + 0.3 wt% ML powders	252 ± 200	450 ± 226
TOPUF + 0.6 wt% ML powders	250 ± 197	390 ± 260
TOPUF + 0.9 wt% ML powders	178 ± 130	257 ± 143
TOPUF + 1.2 wt% ML powders	136 ± 75	282 ± 65
TOPUF + 1.5 wt% ML powders	196 ± 153	278 ± 172

**Table 5:** Average sound absorption and transmission loss of TOPUF adding different forms of ML.

Name	Average Sound Absorption Coefficient	Average Transmission Loss (dB)
PPUF	0.516	10.007
TOPUF	0.422	24.546
TOPUF + 10 cm ML strips	0.492	16.864
TOPUF + 7 cm ML strips	0.478	18.224
TOPUF + 4 cm ML strips	0.487	22.824
TOPUF + 0.9 g ML powders	0.498	20.974

**Table 6:** Comparison of mechanical properties of several PUF.

Name	Compressive Strength (kPa)	Breaking Strength (kPa)	Elongation at Break
PPUF	11.44	41.67	25.93%
TOPUF	56.67	78.33	8.47%
TOPUF + 0.3 wt% ML powders	100.67	128.33	10.31%
TOPUF + 0.6 wt% ML powders	124.44	145.33	7.70%
TOPUF + 0.9 wt% ML powders	133.22	171.67	6.66%
TOPUF + 1.2 wt% ML powders	183.22	156.67	4.58%
TOPUF + 1.5 wt% ML powders	194.56	273.33	9.51%
